# Prevalence and genotypic frequency of color vision defects among primary schoolchildren in Adama Town, Eastern Ethiopia

**DOI:** 10.1186/s12887-024-04529-0

**Published:** 2024-01-22

**Authors:** Temesgen Bedassa Gudeta, Tiruneh Asrat

**Affiliations:** Department of Biology, College of Natural and Computational Sciences, Madda Walabu University, PO Box: 247, Robe, Ethiopia

**Keywords:** Congenital color vision deficiency, Ethnic group, Genotypic frequency, Prevalence, Primary schoolchildren

## Abstract

Color vision deficiency is a common X-linked genetic disorder affecting the day-to-day lives of individuals, in which school-aged children’s academic performance can be negatively affected. The aim of this study was to evaluate the prevalence and genotypic frequency of congenital color vision defects (CVD), among primary schoolchildren in Adama, Ethiopia. A school-based cross-sectional study design was used. Students were purposively selected based on their ethnicity but were randomly selected from their sections, resulting in a final sample size estimated at 846 schoolchildren who had received informed consent from their families. Data was gathered using the Ishihara color vision test, 38-plate edition. The result of the study revealed that the total prevalence of CVD was much higher (5.6%) among the male children than that of the females, which was only about 1.79%. The prevalence rates of CVD among the targeted ethnic groups were found to be the highest among Amhara (7.45%) > Oromo (5.00%) > Gurage (2.13%) children, respectively, in descending order. 62.76% of the study subjects were homozygous dominant (*AA*), followed by those with a heterozygous genotype (*Aa*) (32.51%), and the remaining 4.73% had recessive (*aa*) genes.

## Introduction

Color Vision Deficiency (CVD) is the reduced ability to see color differences under normal lighting conditions. People who do not have such a disorder can visibly distinguish different color bands and are trichromatic, meaning they see a mixture of red, green, and blue colors [1, 2]. The changes or the absence of the absorption spectrum of photopigments leads to a color vision defect, which is about 8% in men and 0.4% in women [3]. Screening of color in humans depends on the unequal stimulation of red, green, and blue cone types by lights of different wavelengths [4].

CVD is an X-linked genetic disorder caused by mutations in the genes encoding the long-, medium-, and short-wavelength-sensitive cone pigments responsible for [5]. Clinically, there are three types of color vision defects: Protan, deuteran, and tritan, with protan and deuteran defects caused by recessive mutations of genes on the long arm of the X chromosome within the Xq28 band [6, 7]. Tritan defects are caused by mutations of the gene encoding blue retinal cone pigment, present on the autosomal chromosome number seven [3, 7].

Hereditary CVD is one of the most widely investigated genetic markers in the study of individuals’ genetic variations in the area of population genetics. The molecular nature of normal red-green color vision pigments and their defects were first revealed in 1986. Males with normal color vision have one red pigment gene and one or more green pigment genes [2, 4]. Red-green color vision defects from mild to severe are formed due to homologous recombination between red and green pigment genes, leading to deletions in green pigment genes or full-length hybrid or fusion genes [5, 7]. Visual problems including CVD can negatively affect social interactions and learning. Studies have shown that, approximately 75–90% of classroom learning is acquired through the visual pathway, either wholly or partially [2, 5, 8, 9].

Estimating the CVD phenotypes and gene frequencies among different populations has several benefits in occupations, jobs, and routine life that involve precise color matching [5]. These include telecommunication and electrical mechanics, seamen, train drivers, air traffic controllers, painters, and several other jobs, as well as daily routine work deemed to require color recognition [3, 5]. As the visual medium is used more in teaching–learning activities, children with color vision defects may be at a disadvantage when compared with normal children [2, 6, 10].

Although there is no treatment for such genetic defects, studies have shown that early diagnosis of these defects helps children better adapt to school tasks and adults understand their limitations at work [3, 6, 9, 10]. The society in the study area, including students and teachers of primary schools, had not yet heard about color vision deficiency and its negative sides. Therefore, the present study aimed to determine the prevalence and genotypic frequency of color vision deficiency among school-age children of various ethnic groups in Adama town, Ethiopia.

## Materials and methods

### Study area description

The study was carried out among three purposefully selected primary schools in Adama town, Ethiopia, namely Burka Boku, Gada Robale and Adama Bosat (Table 1). Adama town is located in the Rift Valley, on flat land with mountains and ridged topography surrounding it. Adama city is located 100 km southeast of Addis Ababa, about 8025′00" and 8036′00" North Latitude and 39,011′ 57" to 39021′15" East Longitude at an average altitude of 1620 m above the mean sea level [11].

**Table 1:** Proportional distribution of sample size across the study primary schools of Adama town

N0	Targeted primary school	Total population	Selected sample size	Male	Female
1	Burka Boku primary school	5594	351	282	69
2	Geda Robale primary school	5012	315	252	63
3	Adama Boset primary school	2849	180	144	36
	Total	13455	846	678	168

Source: [12]

### Study design and study population

A school-based cross-sectional study design was used to determine the prevalence and genotypic frequency of color vision defects among three primary schools in Adama town, Ethiopia, where the study population included both male and female students of Oromo, Amhara and Gurage ethnic groups from grades 3 to 8, with an age range of 9 to 16 years, who are able read letters and numbers (Table 1).

### Inclusion and exclusion criteria

Regarding the inclusion criterion, all voluntary children from Gada Robale, Burka Boku, and Adama Bosat primary schools, from Adama town, with the ethnicities of Oromo, Amhara, and Gurage who had written consent from their parents or adult guardians were included. Students with normal sight participated in the test for a colour vision defect. Students who did not give full cooperation and had not received written consent from their parents, who were on leave during data collection, who had difficulty in communication, who usually wore eyeglasses and blind students were also excluded. Besides this, students whose ethnicity was other than Oromo, Amhara, and Gurage were not included, because of their number was not significant to be studied.

### Sample size determination

To determine the sample size, a simple proportional formula was employed for sample size determination, considering that the previous recent study on the prevalence of color blindness in Ethiopia estimated it at 4.2% [2, 13]. Therefore, *P* = 0.042 and q = 1–0.042 = 0.958 at the 95 CI (confidence interval) and assuming that margin of error is 2% (0.02).

N = sample size; p = proportion of color blindness (0.042); d = margin of error of 2% (0.02).

Q = 1-p = 1-0.042 = 0.958

Z = 1.96 at 95% CI

$$n=\frac{Z{ }^{2}pq}{d{ }^{2}}$$

Hence, using the above formula,

$$n=\frac{{\left(1.96\right)}^{2}\left(0.042\right)\left(0.958\right)}{{\left(0.02\right)}^{2}}=\frac{0.15457}{0.0004}=386$$

To avoid the non-response rate, 10% of the actual sample size (39) was added. Hence, the total sample size was 386 + 39 = 425. Considering the design effect of two and equal participation of the three ethnic groups (Oromo, Amhara, and Gurage) in each of the three primary schools (Table 2), 846 study subjects participated in the study. The actual total sample size (846) was proportionally distributed to each of the three selected primary schools (Table 1) using the following formula.

$$n=\frac{\mathrm{Total}\;\mathrm{sample}\;\mathrm{size}\;X\;\mathrm{total}\;\mathrm{number}\;\mathrm{of}\;\mathrm{students}\;\mathrm{at}\;\mathrm{the}\;\mathrm{school}}{\text{N}}$$

Where N is the total of students in all targeted primary schools.

**Table 2:** Participation of different ethnic groups across the study primary schools of Adama town

N0	Targeted primary school	Population	Sample size selected	Male	Female
1	Burka Boku primary school *n* = 351	Oromo	117	94	23
		Amhara	117	94	23
		Gurage	117	94	23
2	Geda Robale primary school *n* = 315	Oromo	105	84	21
		Amhara	105	84	21
		Gurage	105	84	21
3	Adama Boset primary *n* = 180	Oromo	60	48	12
		Amhara	60	48	12
		Gurage	60	48	12
	Total		846	678	168

### Sampling technique

Each proportionally allocated number (351 for Burka Boku, 315 for Gada Robale, and 180 for Adama Boset Primary Schools, Table 1) was equally assigned to the three ethnic groups to allow them to equally participate, Table 2. The students were purposefully selected for their ethnicity but randomly sampled from their sections (classes) until their number reached the determined sample size. For example, as indicated in Table 2, in Burka Boku Primary School to get 351 participants, equal number of students (117) were randomly selected from each ethnic group. The ethnicity of the student participants was confirmed by the information gained from them and their guards based on their willingness. Since hereditary CVD is much more prevalent in males (8%) than in females (0.4%) [2, 3, 13–15], sex-wise selection of the students was carried out by taking a greater number of males (nearly 80%) to get the case CVD. As indicated hereunder, in Table 2, 678 is nearly 80% of the total sample size, 846.

### Data collection

The data about socio-demographic characteristics were collected in a face-to-face interview using structured questionnaires prepared in the English language and then translated to Amharic by the language expert for the participants. Amharic language is the common local language for the ethnic groups and the society in Adama town. All the student participants were able to communicate in Amharic language very well. After filling out the prepared consent letter format the data for the socio-demographic characteristics was collected and then all the study subjects were evaluated for CVD. Color vision test was done using the Ishihara test pseudoisochromatic plates (38 plates edition) as per [2, 3]. Before performing the test, the purpose and procedure were clearly explained to all subjects. Then, the test was conducted in a room with adequate natural daylight. The plates were held 75 cmfrom the study subject and the schoolchildren were asked to read the numerals with the assistance of two eye experts. The results were classified based on the instructions described in the attached manual of Ishihara’s test plates. Overall, the test was carried out based on the standard recommendation of the colour vision test. The data was collected by three eye experts. Data quality control was employed via training of data collectors, supervision of data collection and the questionnaire was pretested.

### Data analysis

The socio-demographic data were recorded into a computer on an Excel sheet and subsequently transfer to statistical software and then export to SPSS (version 21) for analysis. Descriptive statistics were used to present the socio-demographic characteristics of the study participants. The collected data was manually cleaned and checked. Frequency distributions and chi-square (χ2) test are used to assess statistical significance and *p*-values of less than 0.05 are considered as statistically significant.

$${x}^{2}=\sum \frac{{\left({\text{O}}-{\text{E}}\right)}^{2}}{E}$$

Where O = Observed number, E = expected number.

### Determination of allelic and gene frequency

To determine the allelic and gene frequency of the identified color vision deficiencies, the Hardy–Weinberg equation (p^2^ + 2pq + q^2^ = 1) was used. For genetic data analysis, the phenotypes were recorded for color blindness for each individual and the allele frequencies were calculated according to Hardy–Weinberg law using a gene counting method. Considering *A* allele = p, and *a*n allele = q, then gene frequencies for color blindness were calculated by Hardy Weinberg method (p^2^ + 2pq + q^2^ = 1). The allelic frequency of CVD among male and female students was also determined with the use of a simple gene frequency determination calculation for male, female, and combined sexes as follows.

i) For male: $a=\frac{\%\;\mathrm c\mathrm o\mathrm l\mathrm o\mathrm r\;\mathrm b\mathrm l\mathrm i\mathrm n\mathrm d\;\mathrm p\mathrm e\mathrm n\mathrm o\mathrm t\mathrm y\mathrm p\mathrm e}{100}$, Then, *A* = 1-*a* ---------- [3].

ii) For female: $a=\frac{\sqrt{\%\;\mathrm c\mathrm o\mathrm l\mathrm o\mathrm r\;\mathrm b\mathrm l\mathrm i\mathrm n\mathrm d\;\mathrm p\mathrm e\mathrm n\mathrm o\mathrm t\mathrm y\mathrm p\mathrm e}}{100}$, Then, *A* = 1-*a* ------- [3].
iii) For combined group: $A=\frac{1}{3}a\left(Male\right)+\frac{2}{3}a\left(Female\right)$, Then, *A* = 1-*a* ------- [3].

The homozygosity (Ho) and heterozygosity (Ht) were determined using the formula [3]:

$${H}_{o}= {\Sigma Pi}_{2}$$

Where H_o_ is the homozygosity of the allele and Pi represents the alleles (*A* or *a*). Then H_t_ = 1- Σ H_o_.

## Results

### Socio-demographic characteristics

A total of 846 study subjects (678, or 80.14% males and 168, or 19.86% females) participated in the present study from three ethnic groups chosen from three purposely selected primary schools for color vision deficiency with a response rate of 100%. The majority of the participants were, aged 9–12 years (54.26%). Only 6 (0.71%) of the subject children were found above 16 years old of which (33.33%) were with the CVD case. Out of the examined 846 schoolchildren, the majority [525(62.05%)] of them participated from the 5–8 grade level, Table 3. Similarly, out of 74 female schoolchildren in the age interval of 13–16 only one (1.35%) CVD case was observed whilst almost all [15 out of 16 (93.75%)] of the CVD cases in the same age interval were male. Most (79.52%) of the subjects in the age interval of 9–12 were male whereas the rest 20.48% were female students, Table 3.

**Table 3:** Socio-demographic characteristics and distribution of primary school children in Adama town, Ethiopia

Variables	Description	Condition of Color vision		Total n (%)	*X*^2^	df	*p*-value
		Normal n (%)	Defected n (%)				
Sex:	Male	640 (94.4)	38 (4.49)	678 (80.14)	3.14	1	*P* < 0.021
	Female	165 (98.21)	3 (0.35)	168 (19.86)			
Age:	9–12	M = 344 (94.25)	21 (5.75)	459 (54.26)	4.01	2	*P* < 0.051
		F = 92 (97.87)	2 (2.13)				
		T = 436 (95.0)	23 (5.0)				
	13–16	M = 292 (95.11)	15 (4.89)	381 (45.03)			
		F = 73 (98.64)	1 (1.36)				
		T = 365 (95.80)	16 (4.20)				
	Above 16	M = 4 (66.67)	2 (33.33)	6 (0.71)			
		F = 0 (00.0)	0 (0)				
		T = 4 (66.67)	2 (33.33)				
Grade level:	3–4	M265 (95.32)	13 (4.68)	321 (37.95)	3.41	1	*P* < 0.047
		F = 43 (100.0)	0 (0.0)				
		T = 308 (95.95)	13 (4.05)				
	5–8	M = 375 (93.75)	25 (6.25)	525 (62.05)			
		F = 122 (97.60)	3 (2.40)				
		T = 497 (94.67)	28 (5.33)				
School Name:	Burka Boku	M = 265 (94.0)	17 (6.0)	351 (41.50)	5.92	2	*P* < 0.043
		F = 68 (98.55)	1 (1.45)				
		T = 333 (94.87)	18 (5.13)				
	Gada Robale	M = 239 (94.84)	13 (5.16)	315 (37.23)			
		F = 62 (98.41)	1 (1.59)				
		T = 301 (95.56)	14 (4.44)				
	Adama Bosat	M = 136 (94.44)	8 (5.56)	180 (21.27)			
		F = 35 (97.22)	1 (2.78)				
		T = 171 (95.0)	9 (5.0)				
Ethnic Group:	Oromo	M = 214 (94.70)	12 (5.30)	282 (33.33)	3.32	2	*P* < 0.001
		F = 54 (96.43)	2 (3.57)				
		T = 268 (95.0)	14 (5.0)				
	Amhara	M = 206 (91.15)	20 (8.85)	282 (33.33)			
		F = 55 (98.21)	1 (1.79)				
		T = 261 (92.55)	21 (7.45)				
	Gurage	M = 220 (97.35)	6 (2.65)	282 (33.33)			
		F = 56 (100)	0 (0)				
		T = 276 (97.87)	6 (2.13)				

*M* Male, *n* Frequency of the sample, *F* Female, *T* Total*, X*^2^ Chi-square, *df* Degree of freedom, *M* Male, *F* Female, *T* Total

### Comparison of study subjects of different ethnic groups across the primary schools based on the prevalence of the screened CVD

Although there was an equal distribution of the three ethnic groups in each primary school, the CVD prevalence was observed differently, Table 4. For example: at Burka Boku primary school each of the ethnic groups was distributed in equal frequency as 33.33% (117/351) but, the prevalence of CVD among children from the Gurage ethnic group was observed as much less than those observed in other two ethnic groups. Moreover, out of the total sampled 117 Oromo male children from Burka Boku primary school only 5.32% (5/94) of them were observed with CVD cases, Table 4.

**Table 4:** Comparison of the targeted ethnic groups across the primary schools of Adama town based on the prevalence of the screened CVD

Ethnic Group	Sex	Primary Schools									Total
		Burka Boku			Gada Robale			Adama Bosat			
		n	CVD	Total	n	CVD	Total	n	CVD	Total	
Oromo	Male	89	5	94	80	4	84	45	3	48	226
	Female	22	1	23	20	1	21	12	0	12	56
	Total	111	6	117	100	5	105	57	3	60	282
Amhara	Male	88	6	94	79	5	84	39	9	48	226
	Female	23	0	23	21	0	21	11	1	12	56
	Total	111	6	117	100	5	105	50	10	60	282
Gurage	Male	91	3	94	82	2	84	47	1	48	226
	Female	23	0	23	21	0	21	12	0	12	56
	Total	114	3	117	103	2	105	59	1	60	282
Sum total		333	18	351	301	14	315	171	9	180	846

*n* Frequency of normal sample, *CVD* Color vision defect

Regarding the ethnic groups, out of the diagnosed students from Adama Bosat primary school, the highest CVD prevalence, 16.67% (10/60) was observed among children from Amhara ethnic group; whereas the lowest CVD prevalence 1.67% (1/60) was recorded among children from Gurage ethnic group of the same primary school. According to this study, there was no CVD prevalence recorded among female children from Gurage ethnic group across all primary schools, Table 4. Based on the combined analysis, Table 5, the prevalence rates of congenital color vision deficiency, CVD, among the targeted ethnic groups were found to be the highest among Amhara (7.45%) > Oromo (5.00%) > Gurage (2.13%) respectively in increasing order. Out of the total tested 168 female children only 3 (1.79%) were obtained with CVD. Accordingly, out of the total identified 3 female CVD cases 2 (66.67%) of them were from Oromo Ethnic group. The highest prevalence rate of CVD (8.85%) was identified among male students of Amhara ethnic group in which twenty male students were found to be with CVD out of 226 sampled students in the ethnic group. Likewise, the lowest CVD prevalence (2.13%) was observed among students from Gurage ethnic group in which there was no observed female with CVD.

**Table 5:** Prevalence rates and phenotypic frequency of color vision defects among students of different ethnic groups from primary schools in Adama town, Eastern Ethiopia

Ethnic groups	Combined (both male and female)			Male			Female		
	n (%)	CVD (%)	Total	n (%)	CVD (%)	Total	n (%)	CVD (%)	Total
Oromo	268 (95.0)	14 (4.96)	282	214 (94.70)	12 (5.30)	226	54 (96.43)	2 (3.57)	56
Amhara	261 (92.55)	21 (7.44)	282	206 (91.15)	20 (8.85)	226	55 (98.21)	1 (1.79)	56
Gurage	276 (97.87)	6 (2.12)	282	220 (97.35)	6 (2.65)	226	56 (100)	0 (0)	56
Sum total	805 (95.15)	41 (4.84)	846	640 (94.40)	38 (5.60)	678 (80.0)	165 (98.21)	3 (1.79)	168 (20.00)

Results presented in parenthesis are in %

*n* Number of Normal individuals, *CVD* Color vision deficiency

**Fig. 1:**
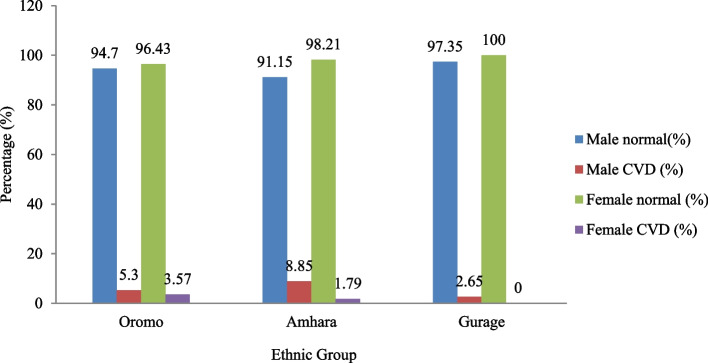
The prevalence of CVD among the targeted three ethnic groups of primary schools in Adama town (every number on the top of every graph represents the prevalence of CVD in %)

Generally, in this study, the prevalence of CVD was higher among the 678 males who account for about 4.49% with a proportion of 38/846 than 168 female students which are only about 0.35%) with the proportion of 3/846 as illustrated in Fig. 1.

### Awareness status of screened CVD primary schoolchildren

Out of the screened 41 schoolchildren from the three primary schools of Adama town, it was confirmed that none of them was with knowledge of the idea of CVD. Although few (19.51%) of the screened children recognized they had eye-related problems, there was no child who reported that she/he had undergone color vision examination at least once in her/ his lifetime, Table 6.

**Table 6:** Awareness status of screened CVD primary schoolchildren in primary schools of Adama town, Eastern Ethiopia

Questionnaire types	Yes		No	
	Frequency	percentage	Frequency	percentage
Do you know the idea of color blindness?	0	0	41	100
Do you have any eye-related problems?	8/41	19.51	33/41	80.49
Have you ever checked up your color vision at least once in your lifetime?	0	0	41	100
Do you have difficulty differentiating various colors?	4/41	9.75	37/41	90.25
Is there a family member who is/are in a case of color vision problem?	0	0	41	100

### The type and prevalence of congenital color vision defects (CVD)

In this study, the types of identified congenital CVD using Ishihara color vision test plates were protan-deutan types of congenital color vision defects and those who couldn’t totally identify color (achromatic) Table 7. Accordingly, from the total 41 cases 40 students were found to have congenital protan-deutan (red-green deficiency) and only one boy (from Adama Bosat primary school of Gurage ethnic group) was found to have achromatic type of CVD. Moreover, of the detected schoolchildren having Achromacy, protan and deutan types of congenital defects in male children were 1(0.15%), 16(2.36%) and 21(3.1%) respectively whereas there were no observed achromatic female children, rather a protan and a deutan were found each in 0.6% and 1.2% proportion respectively, Table 7.

**Table 7:** School-wise phenotypic frequency of affected male and female subjects and the type of CVD cases among them in primary schoolchildren of Adama town, Ethiopia

Sex	Schools	n	Achromacy %	Protan %	Deutan %	Total
Male	Burka Boku	282	0 (0.0)	7 (2.48)	10 (3.55)	17
	Gada obale	252	0 (0.0)	6 (2.38)	7 (2.78)	13
	Adama Bosat	144	1 (0.69)	3 (2.1)	4 (2.78)	8
Total		678	1 (0.15)	16 (2.36)	21 (3.1)	38
Female	Burka Boku	69	0 (0.0)	1 (1.45)	1 (1.45)	1
	Gada Robale	63	0 (0.0)	0 (0.0)	1 (1.59)	1
	Adama Bosat	36	0 (0.0)	0 (0.0)	0 (0.0)	1
Total		168	0 (0.0)	1 (0.6)	2 (1.2)	3
Sum total		846	1 (0.12)	17 (2.01)	23 (2.72)	41 (4.84)

n represents the number of tested subjects; Results presented in parenthesis are in %

Concerning the ethnic group-based phenotypic frequency, the maximum CVD case (10/226 or 4.43%) was observed as deutan followed by protan among male schoolchildren of the Amhara ethnic group, Table 8.

**Table 8:** Ethnic group-wise phenotypic frequency of affected male and female subjects and the type of CVD cases among them in primary schoolchildren of Adama town, Ethiopia

Sex	Ethnic group	n	Achromacy %	protan%	Deutan %	Total
Male	Oromo	226	1 (0.44)	4 (1.77)	7 (3.10)	12 (5.31)
	Amhara	226	2 (0.88)	7 (3.10)	10 (4.43)	19 (8.41)
	Gurage	226	1 (0.44)	2 (0.88)	4 (1.77)	7 (3.10)
Total		678	4 (0.59)	13 (1.92)	21 (3.10)	38 (3.10)
Female	Oromo	56	0 (0.00)	1 (1.45)	1 (1.45)	2 (3.57)
	Amhara	56	0 (0.00)	0 (0.00)	1 (1.59)	1 (1.79)
	Gurage	56	0 (0.00)	0 (0.00)	0 (0.00)	0 (0.00)
Total		168	0 (0.00)	1 (0.60)	2 (1.2)	3 (1.79)

n represents the number of tested subjects; Results presented in parenthesis are in %

### Ethnic-based determination of the allelic frequency of CVD

Genotypic frequency among the schools was performed by using the formula of population genetics derived by Hardy and Weinberg, p^2^ + 2pq + q^2^ = 1, where p^2^ is the frequency of the homozygous dominant genotype (*AA*), q^2^ is the frequency of the homozygous recessive genotype (*aa*) and 2pq is the frequency of heterozygous genotype (*Aa*). Accordingly, using the equation, the frequencies of both alleles (allele *A* and allele *a*), genotypes (*AA, Aa and aa*) and the total number of students with homozygous and heterozygous genotypes from the examined total students from different ethnic groups in each targeted school were determined. Applying the above Hardy and Weinberg formula of population genetics, allele and gene frequency for all schools was calculated separately, Table 9. The frequency of the genotype *aa* which means q2 was first calculated since it refers to the affected (CVD) individuals and then the value for q was obtained by square rooting of q2. Once the q value is known, the value for p was obtained by subtracting it from one, since the sum of p and q equals 1. Finally, the frequency of heterozygous genotype, 2pq (*Aa*), was calculated by simple multiplication. Frequency of CVD = Number of CVD individuals divided by the total population. For example, to find the frequency of CVD-affected cases among the tested Oromo primary schoolchildren (Table 5): q2 = 14/282 = 0.05; this is the frequency of the genotype *aa.* In this case, the frequency of the mutant genotype *aa* must be calculated first, because of the CVD-affected cases were due to this gene*.* Eventually, the frequency of the allele *a* was calculated as q =$\surd 0.05$  = 0.22; then to find the frequency *A* allele represented by p the Hardy and Weinberg equation p + q = 1, p + 0.22 = 1; *p* = 0.78. Likewise, it was calculated for the rest ethnic groups across the primary schools.

**Table 9:** Allelic frequency of CVD among primary schoolchildren of Adama town

Targeted ethnic groups					
Oromo		Amhara		Gurage	
Allele	Frequency	Allele	Frequency	Allele	Frequency
*A* (p)	0.78	*A* (p)	0.73	*A* (p)	0.86
*a* (q)	0.22	*a* (q)	0.27	*a* (q)	0.14
Total	1	Total	1	Total	1

### Sex-based allelic frequency of CVD among primary schoolchildren of different ethnic groups in Adama town, Ethiopia

The allelic frequency of CVD among male and female students was also determined with the use of a simple gene frequency determination calculation for male, female and combined sexes as follows.

For males: $a=\frac{\%\;\mathrm C\mathrm V\mathrm D\;\mathrm i\mathrm n\;\mathrm m\mathrm a\mathrm l\mathrm e\;\mathrm p\mathrm h\mathrm e\mathrm n\mathrm o\mathrm t\mathrm y\mathrm p\mathrm e}{100}$; then *A* = 1-*a*. -------------------- (α)

Then depending on the figures in Table 5, 5.30% CVD for Oromo male children phenotype and incorporating it into the above formula (α) the frequency of recessive allele *a* was calculated as

$$a=\frac{5.30}{100}=\underline{0.053}$$

For female: $a=\frac{\sqrt{\mathrm{CVD}\;\mathrm{female}\;\mathrm{phenotype}}}{100}$; then *A* = 1-*a*. --------------------(β)

Similarly, using values in Table 5, 3.57% CVD female phenotype among students of the Oromo ethnic group, √3.57 divided to 100 the frequency of recessive allele *a* was calculated as

$$a=\frac{\sqrt{3.57}}{100}=\underline{0.019}$$

For the combined group: $\frac13Xa\left(in\;male\right)+\frac23Xa\left(in\;female\right)$; then *A* = 1-*a*. ------------- (µ)

Hence, based on the above calculations, it was obtained that the frequency for allele *a* (recessive allele) among students of each ethnic group was estimated, Table 10.

**Table 10:** Sex-based allelic frequency of CVD among primary schoolchildren of different ethnic groups in Adama town

Ethnic groups	Allele	Frequency		
		Male	Female	Combined
Oromo	*a*	0.053	0.019	0.030
	*A*	0.947	0.981	0.970
Amhara	*a*	0.089	0.013	0.038
	*A*	0.911	0.987	0.962
Gurage	*a*	0.027	0.000	0.009
	*A*	0.973	1.000	0.991

Accordingly, the allelic frequency for recessive allele *a* was more prevalent in boys than in girls in all the studied ethnic groups because of the reason that CVD is more prevalent in boys about their single X-chromosome. The frequency of recessive allele *a* showed a slight difference among the boys of the three ethnic groups with the highest value among boys of Amhara (0.089) and the lowest value (0.027) among the Gurage ethnic groups, Table 10. Likewise, the frequency of the recessive allele *a* was highest to be exhibited among female students of the Oromo ethnic group (0.019) followed by that of the Amhara ethnic group (0.013). However, it was not observed (*i.e.:* zero) among female students of Gurage ethnic groups as stated in Table 10.

### Genotypic frequency and heterozygosity condition of CVD among primary schoolchildren of different ethnic groups in Adama town

Here under, Table 11 presents the genotypic frequencies among male and female children of the three different ethnic groups from primary schools Adama town. It was calculated using the Hardy–Weinberg equation (p2 + 2pq + q2 = 1). Since the male is with single X chromosome, the genotypic frequencies are the same as the allelic frequencies (allele *A* and allele *a*). The females with two X-chromosomes present the three types of genotypes: homozygous dominant (*AA*), homozygous recessive (*aa*) and heterozygous (*Aa*) in which their genotypic frequency was calculated p2, 2pq and q2 respectively. There was 100% observed homozygous *AA* genotypic frequency among female students of the Gurage ethnic group. The heterozygous genotype (*Aa*) was found to be the highest (3.73%) among female children of the Oromo ethnic group while there was no observed child with the heterozygous genotype *Aa* from female children of the Gurage ethnic group, Table 11.

**Table 11:** Genotypic frequency distribution among males and female of different ethnic groups from primary schools of Adama town, Ethiopia

Ethnic groups (population)	Genotypes in Male		Genotypes in Female		
	*A* (Y)	*a* (Y)	*AA* (p2)	*Aa* (2pq)	*aa* (q2)
Oromo	0.947	0.053	0.96236	0.03728	0.00036
Amhara	0.911	0.089	0.97417	0.02566	0.00017
Gurage	0.973	0.027	1.00	0.000	0.00000

In males, *A* (Y) and *a* (Y) present homozygous dominant and homozygous recessive genotypes respectively, where Y represents the Y-chromosome. In females, *AA*, *Aa* and *aa* represent the homozygous dominant, heterozygous and homozygous recessive genotypes, respectively. The total number of individuals with the homozygous and heterozygous genotypes in each studied targeted ethnic group was also determined by multiplying the gene frequency values of each genotype with the total number of students examined in each ethnic group (Table 12). The number of individuals for a given genotype was calculated by multiplying the frequency of each allele from Table 9 by the total number of students in each ethnic group.

**Table 12:** Genotypic frequencies and the total number of students of different ethnic group from primary schools of Adama town, Ethiopia

Ethnic group	Genotype	Frequency (From Table 9)	Total number of individuals	%
Oromo	*AA* (p2)	0.78 × 0.78 = ~ 0.61	0.61 × total population (282) = 172.07 = ~ 172children	61
	*Aa* (2pq)	2 × 0.78 × 0.22 = ~ 0.34	0.34 × total population (282) = 95.88 = ~ 96 children	34
	*aa* (q2)	0.22 × 0.22 = ~ 0.05	0.05 × total population (282) = 14.1 = ~ 14 children	5
	Total	1	324	100
Amhara	*AA* (p2)	0.73 × 0.73 = ~ 0.533	0.533 × total population (282) = 150.2 = ~ 150	53.3
	*Aa* (2pq)	2 × 0.73 × 0.27 = 0.3942	0.394 × total population (282) = 111.16 = ~ 111 students	39.4
	*aa* (q2)	0.27 × 0.27 = ~ 0.073	0.073 × total population (282) = 20.59 = ~ 21 students	7.3
	Total	1	282	100
Gurage	*AA* (p2)	0.86 × 0.86 = ~ 0.74	0.74 × total population (282) = 208.68 = ~ 209 students	74
	*Aa* (2pq)	2 × 0.86 × 0.14 = 0.2408	0.24 × total population (282) = 67.68 = ~ 68 students	24.1
	*aa* (q2)	0.14 × 0.14 = 0.019	0.019 × total population (282) = 5.3 = ~ 5 students	1.9
	Total	1	282	100
	Sum total	1	846	100

Using the calculated allelic frequencies of CVD among different ethnic groups of primary schoolchildren explained in Table 9, above, and the calculated sample size of each ethnic group, the total number of the schoolchildren with pure homozygous dominant (*AA*), heterozygous dominant (*Aa*) and pure recessive (*aa*) genotype was determined. Accordingly, out of the 282 sampled children from Oromo ethnic groups, 61% *i.e.,* 172/282 of them exhibited pure homozygous dominant (*AA*) genotype, whereas only 5% was with pure recessive (*aa*) genotypes, Table 12. Similarly, there were 53% (149/282) of students from the Amhara ethnic group exhibited pure homozygous dominant (*AA*) genotype which is less comparable with that observed among students of the Oromo ethnic group. As it is indicated in Table 9 among students of the Gurage ethnic group the allelic frequency for the dominant allele *A* was estimated as 0.86 which in turn generated 0.74 (74%) pure homozygous dominant (*AA*) genotype, Table 12. Therefore, though all the ethnic groups participated with equal proportions (282/846) in this study, the highest proportion (209/282) of pure homozygous dominant (*AA*) genotype was recorded among students of Gurage ethnic groups. This indicates there is less availability of carrier genes (only 2%) among students of Gurage ethnic groups when compared to the others *i.*e. (*AA*) genotypes from Gurage (209/282) > (*AA*) genotypes from Oromo (172/282) > (*AA*) genotypes from Amhara (149/282).

**Fig. 2:**
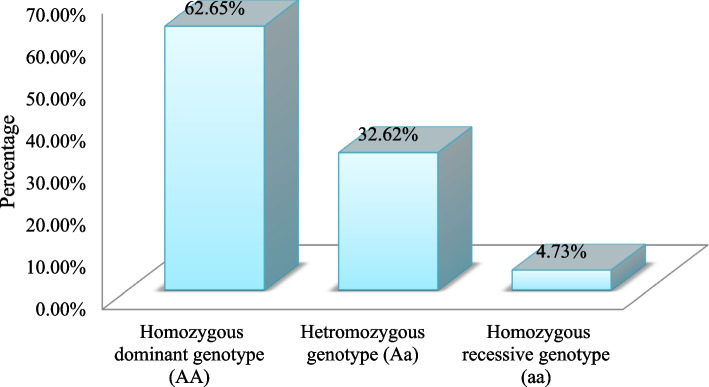
Percentage and types of observed genotypes in 846 sampled schoolchildren of Adama town, Eastern Ethiopia

Generally, out of the studied subjects of all three ethnic groups, 530/846 (62.65%) of them were with pure homozygous dominant genotype (*AA*), followed by those with heterozygous genotype (*Aa*) 276/846 (32.62%) whereas the least remaining 40 (4.73%) were children with pure recessive (*aa*) gene, Fig. 2.

The total prevalence of CVD, in this study, was found to be 4.84% which was much higher among the school male children (38 out of 678) when compared to that of females which was only about 1.79% (3 out of 168), Table 5.

## Discussion

In general, the occurrence of congenital color vision deficiency, in this study, is severely widespread among males but rare in females which is in line with the studies made in Ethiopia and other countries on the world, Table 13.

**Table 13:** References indicating distribution of CVD as per the findings of different studies in Ethiopia and different population across the world

References	Place (country) of the conducted research	Finding of the research		
		Male	Female	Total
[3]	Jammu and Kashmir (India)	4.0%	0.43%	4.43%
[16]	Turkey	7.3%	0.17%	7.47%
[15]	Wardha District (India)	3.7%	0.4%	4.01%
[2]	Gurage administrative zone (Ethiopia)	4.20%	0.20%	4.04%
[14]	Wolkite (Ethiopia)	3.60%	0.60%	4.10%
In this study (as indicated in Table 5)	Adama town (Ethiopia)	5.6%	1.79%	4.84%

The prevalence of CVD ranged from 2.65% (in the Gurage ethnic group) to 8.85% (in Amhara ethnic group) among males and 0% (in the Gurage ethnic group) to 3.57% (in the Oromo ethnic group) among female children of the three primary schools, Table 5. In this case, male children tend to have higher CVD frequency which is attributed to the fact of X-linked recessive nature of the trait (i.e., the single X-chromosome in males is predominant to color blindness, while females with two X-chromosomes can act as dosage compensation and decreases the risk of the disease) [2, 6]. Likewise, the congenital CVD was found much more (16.67%) in Amhara ethnic group, but no one from Guragae. This could be due to the difference in their genetic background such that the predisposition of the sampled population from Amhara ethnic group to CVD through their original population (founders), whereas that of Guragae ethnic group were perhaps without the case. On the other hand, there might also be a restricted gene flow among the population of two ethnic groups [14].

The prevalence of CVD, in the present study, was 4.84%; which is nearly comparable with the prevalence rates of previously conducted research: 4.2% in Abeshege district [2] and 4.1% in Wolkite [14] (both from Southern parts of Ethiopia), 4.43% in Jammu and Kashmir of India [3] and 4.2% in Thailand [17]. However, the result of this study is higher than the incidence of color blindness among school children in Kathmandu valley of Nepal at 3.9% [10] and 2.6% in Nigeria [18]. A higher prevalence rate of CVD, 5.3% in Singapore [19], 5.9% in Korea [20] and 7.47% in Turkey [16] was reported. Similarly, the prevalence of red-green color blindness among male children in this study accounts for 5.60% which is far from the findings of studies from other parts of the world such as 8.1% in Iran [21] and 8.7% in Jordan [22] showed greater difference for female children, this value (1.79%) is in line with studies of other parts of Ethiopia by Mulusew and Yilikal [2] and many other countries of the rest of the world which account mostly less than 1% [3, 15]. These variations in the prevalence of CVD from the current study could be due to differences in the study population, variations in the geographical area, ethnicity and techniques of color vision test.

In this study, the highest CVD was identified in male students of Amhara ethnic group. This might be due to their predisposition in their genetic background and males are hemizygous for the CVD trait where the carrier gene from X-chromosome is dominant over that of Y-chromosome [3, 5, 7]. In this regard, if there were *‘*double carriers*’* or compound heterozygote mothers, who had a recessive allele for deutan on one X chromosome, and a recessive allele for protan on the second X chromosome in Amhara ethnic group, the number of sons with CVD can increases as reported by Carroll [23]. Congenital CVD is genetically determined by X-linked recessive inheritance and thus occurs in males but is transmitted via females where it varies among population, races and locations [3, 24]. However, in Oromo ethnic group the highest CVD was obtained among female students may be due to the practice of consanguineous marriage among population of Oromo ethnic group. Furthermore, the molecular structure of gene on the X chromosome may also contribute for the difference in the prevalence of CCVD between populations and geographic regions [24].

There was 100% observed pure homozygous *AA* genotypic frequency among female students in the Gurage ethnic group of the primary schools. The heterozygosity genotype (*Aa*) was found the highest (3.73%) among female children of the Oromo ethnic group, while there was no (zero) observed *Aa* among female children of Gurage ethnic groups. This finding is almost similar to the study carried out by Fareed et al., [3] who studied the genotypic frequency and heterozygosity of color blindness among six populations from the North India region, the Mughal, Malik and Mir regions present 100% of pure dominant genotypic frequency and accounts 0.00% for heterozygous and homozygous recessive genotypes. According to this author, the heterozygosity was found the highest among Syed (28.76%) population, while the least among Gujjar and Bakarwal (19.64%) and Khan (19.14%) and accounts for zero for Mughal, Malik and Mir populations.

## Conclusion

Early testing of CVD has not yet been practiced in Ethiopia including Adama town when students join schools and universities. This study is a kind of diagnostic study to screen congenital CVD individuals among school populations. It is very important for highly populated and multi-ethnic countries like Ethiopia where CVD is prevalent but has not yet been well known. Such screening can help to minimize the risk of transmitting this genetic disorder to the coming generations through preconception or premarital counselling and also through prenatal diagnosis strategies. Diagnosis of these defects early in life helps children adjust better to tasks at school and adults understand their limitations at work. As evidence from this study, the majority of students were unaware of CVD disease. Therefore, there is a need of conducting such indicative study to create awareness about CVD and its impact on various stages of life. Otherwise, the CVD disorder greatly hinders people from choosing certain career opportunities, leading to psychological trauma. Since the prevalence of congenital CVB varies from one ethnic group to the other, such type of ethnicity-based CVD study is significant to compare its prevalence rate among ethnic groups. As a result, the ethnic group with a high prevalence rate of CVD can be helped to minimize the occurrence and the transmission of the disorder among their offspring through parental education and genetic counselling strategies. The confidentiality of the affected CVD children must be respected and their future carrier has to be adjusted. This can minimise the risk factors associated with the disorder. In this study, the CVD of school children was normally diagnosed using Ishihara’s color testing tools. Further studies are important to be conducted by involvement of governmental bodies such as Ministry of Health and Ministry of Education with great concern for better diagnosis of CVD using modern and more advanced technology devises.

## Data Availability

The data used to support the findings of this study are included in Tables 1, 2, 3 and 4 in the same manuscript.
